# Magnesium Supplementation Diminishes Peripheral Blood Lymphocyte DNA Oxidative Damage in Athletes and Sedentary Young Man

**DOI:** 10.1155/2016/2019643

**Published:** 2016-03-06

**Authors:** Jelena Petrović, Dušanka Stanić, Gordana Dmitrašinović, Bosiljka Plećaš-Solarović, Svetlana Ignjatović, Bojan Batinić, Dejana Popović, Vesna Pešić

**Affiliations:** ^1^Department of Physiology, University of Belgrade, Faculty of Pharmacy, 11121 Belgrade, Serbia; ^2^Department of Medical Biochemistry, University of Belgrade, Faculty of Pharmacy, 11121 Belgrade, Serbia

## Abstract

Sedentary lifestyle is highly associated with increased risk of cardiovascular disease, obesity, and type 2 diabetes. It is known that regular physical activity has positive effects on health; however several studies have shown that acute and strenuous exercise can induce oxidative stress and lead to DNA damage. As magnesium is essential in maintaining DNA integrity, the aim of this study was to determine whether four-week-long magnesium supplementation in students with sedentary lifestyle and rugby players could prevent or diminish impairment of DNA. By using the comet assay, our study demonstrated that the number of peripheral blood lymphocytes (PBL) with basal endogenous DNA damage is significantly higher in rugby players compared to students with sedentary lifestyle. On the other hand, magnesium supplementation significantly decreased the number of cells with high DNA damage, in the presence of exogenous H_2_O_2_, in PBL from both students and rugby players, and markedly reduced the number of cells with medium DNA damage in rugby players compared to corresponding control nonsupplemented group. Accordingly, the results of our study suggest that four-week-long magnesium supplementation has marked effects in protecting the DNA from oxidative damage in both rugby players and in young men with sedentary lifestyle. Clinical trial is registered at ANZCTR Trial Id: ACTRN12615001237572.

## 1. Introduction

Modern age has brought upon a life style that is, in young people particularly, accompanied by lack of sleep, imbalanced diet including fast food consumption, excessive amount of stress, reduced physical activity, and alcohol abuse. All these factors contribute to excess inflammation and oxidative stress and exhibit detrimental influence on human genome [1–4]. Each day, the human genome suffers approximately one million lesions, including adducts, modifications, or fragmentation of the sugar phosphate backbone of DNA [5]. If left unrepaired, DNA damage can cause mutations such as base substitutions and chromosomal translocations that disrupt normal gene expression or create abnormal proteins that are detrimental to cellular function or viability [6].

Both endogenous and exogenous factors can increase production of reactive oxygen species (ROS) and induce DNA damage [7]. Endogenous factors include products of cellular metabolism, such as ROS created via mitochondrial oxidative respiration, or produced by lipid peroxidation, and during processes such as phagocytosis. Endogenous damage may also occur due to errors arising from normal cell replication [8, 9]. Exogenous factors, on the other hand, include improper diet, alcohol, cigarette smoking, and environmental toxins [8, 10]. Evidence indicates that oxidative stress induced DNA damage and impaired DNA repair mechanisms are involved in the pathogenesis of cancer, atherosclerosis, neurodegenerative disorders, and chronic lung diseases [11, 12].

Even though physical exercise is considered to have beneficial effects on health, many, but not all studies on this topic, gave evidence that acute and strenuous exercise can induce oxidative stress [13]. Acute and strenuous exercise lead to oxidative stress by excessive production of ROS and reactive nitrogen species (RNS), superoxide (O_2_^∙−^), hydrogen peroxide (H_2_O_2_), hydroxyl radical (OH^∙^), hypochlorous acid (HOCl), and nitric oxide (NO^∙^) that exhibit detrimental effects on macromolecules such as lipids and proteins, but mainly the DNA [13, 14]. During exercise, oxygen consumption can increase up to 10- to 15-fold above resting levels, thus temporarily increasing the rate of mitochondrial free radicals production. Exercise may also induce inflammatory reactions similar to the acute phase response occurring in injury or infection [15].

Rugby matches are considered to be very demanding and cause both physical and psychological stress in participants [16, 17]. Various studies evaluated the changes in immunoendocrine and inflammatory markers, neutrophil function (production of ROS and phagocytic activity), and the recovery time-course of neuromuscular function, concentrations of testosterone and cortisol, mood impairments, and muscle damage after intense competitive physical performance, such as a rugby game [16, 18–21]. Nevertheless, there are few evidences concerning the correlation between rugby-induced stress and the level of oxidative damage [18, 21].

On the other hand, magnesium as an essential element is involved in regulation of cell cycle and apoptosis, stabilizes the structure of nucleic acids [22], protects DNA from alkylation, and has a role as a cofactor of the enzymes of nucleic acid metabolism: it is involved in DNA replication, DNA repair, and gene expression [23, 24].

Having all abovementioned in mind, the aim of this study was to investigate whether four-week-long magnesium supplementation in young men, with either sedentary life style such as students or athletes involved in strenuous exercise such as rugby players, could protect peripheral blood lymphocytes (PBL) from hydrogen peroxide-induced DNA damage evaluated by alkaline Comet test.

## 2. Materials and Methods

### 2.1. Subjects

Twenty-three healthy young male subjects volunteered for this study, thirteen were members of the same amateur rugby team, and the other ten were students of the University of Belgrade with sedentary life style. The participants were divided into four groups, as follows:

 Group 1: students with a sedentary life style, without magnesium supplementation (*n* = 5).
 Group 2: students with a sedentary life style, receiving 500 mg of magnesium per day divided in two doses separated by 12 h interval for 28 days (*n* = 5).
 Group 3: rugby players, without magnesium supplementation (*n* = 5).
 Group 4: rugby players receiving 500 mg of magnesium per day divided in two doses (one tablet of magnesium 250 mg®. Natural Wealth, NBTY Inc.) separated by 12 h interval for 28 days (*n* = 8).

Students had not been involved in any regular exercise for at least six months and they remained sedentary during the whole study. Rugby players trained three to four times a week for 2 hours and played a match on each Sunday in the early afternoon. Three months before and during the study, none of the participants was taking other vitamin or mineral supplements. All participants gave their written consent to participate after being fully informed of all experimental procedures. The investigation was carried out according to the guidelines and study protocol that has been approved by the Ethical Committee for Clinical trials of University of Belgrade, Faculty of Pharmacy, number 199/2.

### 2.2. Anthropometric Data

Rugby players involved in this study were 23.30 ± 0.93 years old and had average height 181.26 ± 1.30 cm, body mass 85.30 ± 2.31 kg, and body mass index (BMI) 25.91 ± 0.51 kg/m^2^ (Mean ± SD). Mean age of students was 22.6 ± 0.52 years, height was 186.9 ± 6.42 cm, body mass was 84.4 ± 6.60 kg, and BMI was 24.16 ± 1.41 kg/m^2^.

### 2.3. Blood Collection

On the 29th day of experiment (first day after 28 days of supplementation) peripheral blood samples were collected from the participants of this study. In the morning from 09.00–10.00 a.m. (about 20 h after the rugby match for athletes), blood was drawn from an antecubital vein in sitting position into 2 mL EDTA Vacutainer tubes and comet assay was performed.

### 2.4. Comet Assay (Single Cell Gel Electrophoresis (SCGE))

The comet assay is highly reproducible, rapid, and sensitive method for measuring DNA damage [25, 26]. The comet test was conducted as described by Singh et al. [25]. Microscope slides were coated with a layer of 1% normal melting point agarose (Sigma-Aldrich, St. Louis, MO) and left to dry on room temperature. The 6 *μ*L of whole blood samples was suspended in 0.67% low melting point (LMP) agarose (Sigma-Aldrich, St. Louis, MO) in phosphate buffered saline (PBS, Torlak Institute of Immunology and Virology, Belgrade, Serbia), applied onto microscope slides (prepared on previously described way) and maintained on 4°C for 5 min, to solidify. Then a second layer of 0.5% LMP agarose was pipetted onto microscope slides. Every sample had a matching positive control that was treated with hydrogen peroxide (1.5 mM H_2_O_2_) and then after 5 min on 4°C with second layer of 0.5% LMP agarose. The following step included immersing all slides in lysing solution (2.5 M NaCl, 100 mM ethylene-diaminetetra-acetic acid, 10 mM Tris at pH 10, 1% Triton X-100 and 10% dimethylsulfoxide, pH 10 adjusted with NaOH) on 4°C and leaving them overnight. After that, DNA in lysed cells was allowed to unwind in alkali buffer (300 mM NaOH, 1 mM EDTA) for 30 min. Samples were then subjected to electrophoresis for another 30 min at 215 mA, 25 V, washed three times (for 5 min each slide) with a neutralizing buffer (0.4 M Tris, pH 7.5), and stained with ethidium bromide (20 *μ*g/mL). Cover slips were then placed on top of microscope slides and DNA damage was visually analyzed. Analyses were performed on Olympus BX 50 microscope (Olympus Optical Co., GmbH, Hamburg, Germany) equipped with a mercury lamp HBO (50 W, 516–560 nm, Zeiss) at 100x magnification.

Cells were graded by eye, as it was described by Anderson et al. [27], into 5 categories based on perceived comet tail length migration and relative proportion of DNA in the comet tail: no damage (<5%, class A); low level of damage (5–20%, class B); medium level of damage (20–40%, class C); high level damage (40–95%, class D); and total damage (>95%, class E). For each blood sample comet assay was conducted on two microscope slides and 100 cells were analyzed (50 cells per slide).

### 2.5. Statistical Analysis

Statistical analysis was performed using SigmaPlot 11.0. Student's *t*-test and Mann-Whitney Rank Sum test were used in order to compare the difference between the groups and *p* values less than 0.05 were considered significant. The results were expressed as mean ± SEM.

## 3. Results

**Figure 1:**
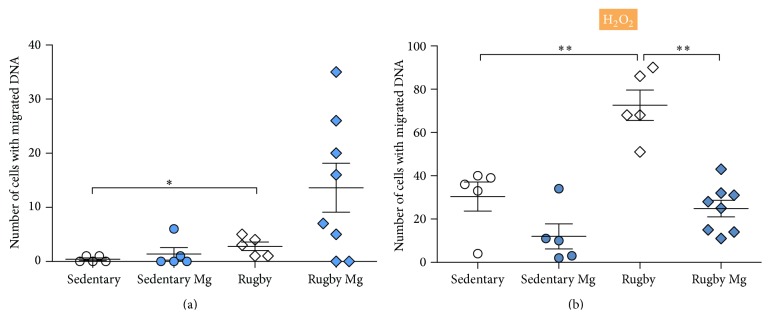
Effects of 4-week magnesium supplementation on number of peripheral blood lymphocytes (PBL) with DNA damage, scored by Comet assay. Participants were divided in four groups in total, due to Mg supplementation and level of physical activity: sedentary, sedentary with Mg supplementation (sedentary Mg), rugby players (rugby), and rugby players with Mg supplementation (rugby Mg). (a) Basal number of cells with migrated DNA. Number of participants per group: sedentary (*n* = 5), rugby (*n* = 5), sedentary Mg (*n* = 5), and rugby Mg (*n* = 8). (b) Number of cells with migrated DNA after exposure to 1.5 mM H_2_O_2_. Number of participants per group: sedentary (*n* = 5), rugby (*n* = 5), sedentary Mg (*n* = 5), and rugby Mg (*n* = 8). Results are shown as means ± SEM. The difference obtained was considered to be statistically significant when *p* < 0.05 (^*∗*^ *p* < 0.05; ^*∗∗*^ *p* < 0.01).

Results from our study show that the level of basal endogenous DNA damage presented as number of cells with migrated DNA (Figure 1(a)) was higher in nonsupplemented rugby players compared to nonsupplemented students (*p* = 0.042). Likewise, in these groups, the levels of DNA damage in cells exposed to H_2_O_2_ were significantly higher in rugby players (*p* = 0.002) (Figure 1(b)).

After four-week-long magnesium supplementation there was no statistically significant difference between students with sedentary lifestyle and rugby players that received supplementation in the total number of cells with damaged nuclei, in the presence of H_2_O_2_ (*p* = 0.080) (Figure 1(b)). However, magnesium supplementation had more pronounced effects in rugby players: athletes that received supplementation had significantly smaller total number of cells with DNA damage, in the presence of H_2_O_2_, compared to rugby players from control group (*p* = 0.002) (Figure 1(b)).

**Figure 2:**
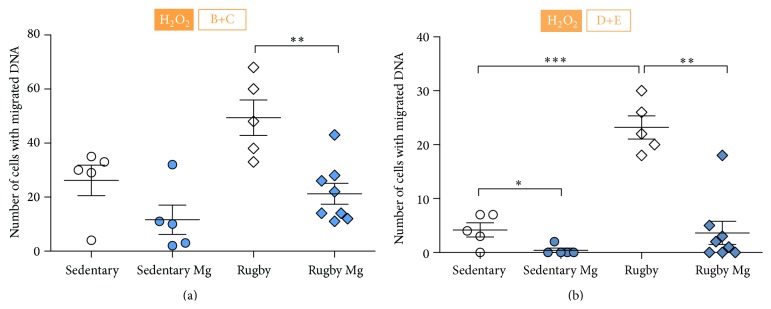
Effects of 4-week magnesium supplementation on the level of DNA damage induced by exposure to 1.5 mM H_2_O_2_ in peripheral blood lymphocytes (PBL), scored by Comet assay. Participants were divided into four groups in total, due to Mg supplementation and level of physical activity: sedentary, sedentary with Mg supplementation (sedentary Mg), rugby players (rugby), and rugby players with Mg supplementation (rugby Mg). (a) Number of cells with low and medium level of DNA damage (B and C). Number of participants per group: sedentary (*n* = 5), rugby (*n* = 5), sedentary Mg (*n* = 5), and rugby Mg (*n* = 8). (b) Number of cells with high and total level of DNA damage (D and E). Number of participants per group: sedentary (*n* = 5), rugby (*n* = 5), sedentary Mg (*n* = 5), and rugby Mg (*n* = 8). The difference obtained was considered to be statistically significant when *p* < 0.05 (^*∗*^ *p* < 0.05, ^*∗∗*^ *p* < 0.01, ^*∗∗∗*^ *p* < 0.001).

Furthermore, the evaluation of degree of DNA damage was done, and scores were divided into low + medium damage and high + total damage and results are presented in Figure 2. There were no significant differences in the degree of DNA damage among groups in the absence of H_2_O_2_. However, after four-week-long magnesium supplementation, the number of cells with low and medium damage (B + C), after exposure to H_2_O_2_, was significantly smaller in rugby players compared to age-matched controls (*p* = 0.002) (Figure 2(a)). Interestingly, this effect was not observed in students compared to age-matched controls (*p* = 0.099) (Figure 2(a)). However, as presented in Figure 2(b), magnesium supplementation was effective in diminishing the number of cells with highly damaged DNA in both students and rugby players compared to respective controls. Results shown in this figure reveal that after H_2_O_2_ exposure the median number of cells with high and total damaged DNA (D + E category) is significantly lower in students and rugby players that received supplementation (*p* = 0.025 and *p* = 0.002, resp.). Also, the number of cells with highly and totally damaged nuclei is significantly higher in rugby players than students in groups that did not receive supplementation (*p* < 0.0001) (Figure 2(b)).

## 4. Discussion

In the present study DNA damage in PBL of rugby players and age-matched group of sedentary young man, university students, was compared after four-week-long Mg supplementation. DNA damage was assessed by alkaline Comet test in the absence or presence of H_2_O_2_. Results of the study showed that PBL of rugby players were more susceptible to DNA damage compared to sedentary young man, both with and without H_2_O_2_ challenge. Furthermore, this study showed that even though Mg supplementation had no effect on DNA damage in unchallenged lymphocytes, it reduced level of high-damaged H_2_O_2_-treated cells in both groups of participants.

Comet assay is used in evaluating changes in DNA integrity such as strand breaks, alkali-labile sites, DNA cross-linking, and incomplete excision repair [28]. Different methods are used nowadays in assessing the level of DNA damage such as high performance liquid chromatography, 8-OH-deoxyguanosine assay, micronuclei test, chromosome aberration, and sister chromatid exchanges. Among them, comet test has a widespread use because of its sensitivity, simplicity, and reliability [29].

In our study, the level of DNA damage expressed as the number of cells with migrated DNA was significantly increased in rugby players on the day after the match compared to sedentary students, indicating that intense exercise exhibits negative effects on DNA integrity. Thus, this study is adding up to data that are proving that strenuous physical activity may lead to DNA instability, since it was demonstrated that acute and strenuous exercise lead to oxidative stress by excessive production of ROS and RNS [13, 14]. Nevertheless, effects of competitive exercise (trainings and matches) on DNA stability, based on the conducted studies, are still controversial. Niess et al. [30] reported an increase in DNA damage in 10 out of 12 participants in a half-marathon 24 h after the race. Hartmann et al. [31] observed similar changes in DNA damage level. Using comet assay, they found elevated DNA migration in six athletes 24 h after a short-distance triathlon which is considered to be an endurance exercise. In both studies, blood was collected and DNA migration was compared 24 h before and after the race. Hartmann et al. [31] took blood samples seven times for the next 5 days and the level of DNA migration remained to be increased 5 days after race. To the best of our knowledge, the majority of studies have shown increased levels of DNA migration 24 h after competitive endurance exercise. However, Briviba et al. [32] found no change in the DNA strand breaks in ten participants, when conducting SCGE assays just after a half-marathon and a marathon race. Nevertheless, different protocols, small number of participants, and differences in the training status of the subjects involved in these studies represent limitations in interpreting the results [30–33].

We have demonstrated that athletes receiving magnesium supplementation for one month had significantly smaller number of PBLs with damaged DNA after H_2_O_2_ treatment, compared to rugby players devoid of supplementation. Magnesium supplementation apparently had protective effects on DNA against oxidative damage in rugby players, since both medium and high level of H_2_O_2_-induced damage were decreased compared to respective levels in nonsupplemented athletes. In addition, this effect was more pronounced in rugby players compared to students in which magnesium supplementation was accompanied by the decrease of DNA damage, but the effect was statistically significant only in decreasing the number of lymphocytes with highly damaged DNA. A possible explanation for the different effect of Mg on PBL DNA damage in rugby players and sedentary young men is the fact that intensive physical activity increases magnesium requirement. Evidence indicates that both short-term high-intensity and long-term strenuous exercise cause significantly increased loss of magnesium through urine and sweat [34]. Also, during exercise, redistribution of magnesium to certain body compartments, with increased energy and ROS production, occurs [34–36]. Therefore, some authors propose that short- and long-term strenuous exercise should be accompanied with 10–20% higher magnesium intake compared to daily intake recommendations for the persons of the same age and sex with sedentary lifestyle [34].

Both animal and human studies indicated that magnesium deficiency has a negative impact on physical performance [34, 37]. However, that is not the only negative consequence of decreased level of magnesium in the body: studies on experimental animals demonstrated that magnesium deficiency leads to swollen mitochondria and disorganized sarcoplasmic reticulum in skeletal muscles, increases formation of lipid radicals and nitric oxide, and impairs endogenous protective mechanisms such as glutathione [38, 39]. It has also been shown that vigorous exercise induces inflammatory response because of tissue injury, consequently increasing the production of ROS, especially in activated phagocytes [15, 40–42]. Takahashi et al. [18] reported no significant change in ROS production in neutrophils right after a rugby game, but they also noticed that 4 h after the end of the game it had increased significantly. Nevertheless, some authors pointed out that chronic moderate exercise may induce adaptive responses in human organism by enhancing the expression of antioxidant enzymes such as Cu/Zn superoxide dismutase (SOD) and by reducing mitochondrial hydrogen peroxide [43, 44] in the muscle tissue. The effectiveness of the adaptive mechanisms in oxidative stress induced by regular physical activity also depends on the individual's lifestyle, nutrition, and expression of genes involved in DNA repair systems [29, 45]. Future studies investigating the effects of strenuous exercise on DNA stability should include longer observation periods and monitor the DNA integrity for at least five days after the match as some authors claim that for major alterations in DNA repair mechanisms take more than 24 hours [15, 33, 46, 47].

Furthermore, it might be taken into account that greater degree of magnesium deficiency which influences antioxidant activity in PBLs makes them more susceptible to oxidative stress caused by hydrogen peroxide, since previous data showed the harmful effect of magnesium deficiency on lipid peroxidation in the cardiovascular system [48], and that tissue homogenates from magnesium deficient animals were more susceptible to lipid peroxidation than animals fed diets adequate in magnesium [48]. Nevertheless, this assumption should be explored and possibly confirmed by estimating level of magnesium in PBLs of rugby players and sedentary students, in the future investigation on the larger population.

Results of this study point to the conclusion that strenuous exercise sensitizes PBLs to oxidative stress and that magnesium supplementation shows protective effects in reducing the level of H_2_O_2_-induced PBL DNA damage thus indicating the importance of adequate magnesium intake in both students with sedentary life style and physically active individuals.
